# Effects of Crowding and Attention on High-Levels of Motion Processing and Motion Adaptation

**DOI:** 10.1371/journal.pone.0117233

**Published:** 2015-01-23

**Authors:** Andrea Pavan, Mark W. Greenlee

**Affiliations:** 1 Universität Regensburg, Institut für Psychologie, Experimental and Clinical Neuroscience Study Programme, Universitätsstr. 31, 93053, Regensburg, Germany; 2 University of Lincoln, School of Psychology, Brayford Pool, Lincoln, LN6 7TS, United Kingdom; University Medical Center Goettingen, GERMANY

## Abstract

The motion after-effect (MAE) persists in crowding conditions, i.e., when the adaptation direction cannot be reliably perceived. The MAE originating from complex moving patterns spreads into non-adapted sectors of a multi-sector adapting display (i.e., phantom MAE). In the present study we used global rotating patterns to measure the strength of the conventional and phantom MAEs in crowded and non-crowded conditions, and when attention was directed to the adapting stimulus and when it was diverted away from the adapting stimulus. The results show that: (i) the phantom MAE is weaker than the conventional MAE, for both non-crowded and crowded conditions, and when attention was focused on the adapting stimulus and when it was diverted from it, (ii) conventional and phantom MAEs in the crowded condition are weaker than in the non-crowded condition. Analysis conducted to assess the effect of crowding on high-level of motion adaptation suggests that crowding is likely to affect the awareness of the adapting stimulus rather than degrading its sensory representation, (iii) for high-level of motion processing the attentional manipulation does not affect the strength of either conventional or phantom MAEs, neither in the non-crowded nor in the crowded conditions. These results suggest that high-level MAEs do not depend on attention and that at high-level of motion adaptation the effects of crowding are not modulated by attention.

## INTRODUCTION

**Fig 1:**
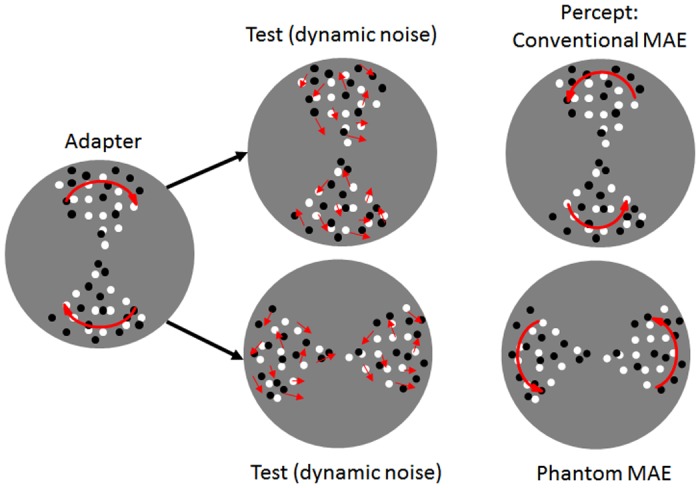
Schematic representation of the stimuli used in the experiments. The 90° angular sectors composing the adapting and test stimuli could be arranged along the vertical axis or the horizontal axis (not shown). Though the horizontal axis is not represented, in the experiments we used both axes. Dots moved globally (100% coherence) either clockwise or counter-clockwise. The red arrows of the adapter represent the (clockwise) motion of the dots within each circular sector. A schematic representation of the dynamic test patterns is also shown. Dynamic test stimuli had a coherence of 0%. The 90° angular sectors composing the test patterns could be arranged along the same axis with respect to the adapting sector, thus inducing a conventional MAE after the motion adaptation period, or could be arranged along the orthogonal axis, inducing a phantom MAE after the adaptation period. See text for more details about stimuli and motion sequence.

Primate single-cell recordings and human neuroimaging investigations have shown that at the highest levels of motion processing neurons encode the global patterns of motion (i.e., optic flow) usually created by forward locomotion through the environment [1–3]. Optic flow components such as radial motion (i.e., contracting/expanding), spiral motion and rotational motion are processed by high-level visual areas such as MT and MST [1, 2, 4–11]. MST neurons are also thought to be involved in high-order forms of motion after-affect (MAE) like the phantom MAE [12–16]. In the phantom MAE, adaptation to some sectors of the visual field that contain, e.g., clockwise, counterclockwise, expanding or contracting motion, subsequently induces the perception of motion in the reversed direction when presenting a test stimulus in non-adapted parts of the visual field. Thus, while in the conventional MAE adapting and test patterns are always spatially overlapping (see [17, 18] for reviews) in the phantom MAE adapter and test patterns are not presented in the same retinal location (see Fig. 1). Phantom MAE is likely to depend on detectors with large receptive fields, like those reported in the dorsal part of the MST (MSTd) in macaque monkeys which span up to 100° of the visual field [3, 19], that are sensitive to various components of object or self-movement and pool a wide range of motion signals across different parts of the visual field [1, 3, 5, 7, 15, 19–26]. In humans, Morrone et al. [7] and Smith et al. [10], using fMRI provided evidence for the presence of distinct cortical areas responding to optic flow components and translational motion. In particular, within the human MT complex (hMT+), it was found that the region that responds to optic flow (e.g., radial motion) was more ventral and approximately 1 cm away from the area responding to translational motion. Adapting their participants to expanding and rotational (clockwise) motion, Wall et al. [11] found fMRI evidence of neural adaptation of optic flow components in area MST. In addition, selectivity to optic flow was found in cortical area MT and V3A, albeit very weak, but not at all in V1, V2, V3 and V4. Moreover, there is neurophysiological and computational evidence that the tuning and selectivity to optic flow components exhibited by MST derives from the integration of nonlinearly transformed local motion signals from MT [7, 27–29]. Mineault et al. [27] developed a hierarchical model of optic flow analysis and showed that a nonlinear integration in the form of compressive nonlinearity [30] is necessary to increase the overall level of responses to optic flow components (e.g., spiral motion and deformation moving stimuli) relative to translational stimuli, while preserving the shape of the neurons’ tuning curves.

In the present study we aimed to investigate the nature of the phantom MAE, assessing the potential roles of awareness and attention in its formation. In order to tap high-level motion analysis observers were adapted to rotational motion (i.e., clockwise and counterclockwise motion). In particular, stimuli consisted of globally moving dots presented in two sectors of a complete circle, with each sector subtending a central angle of 90° [13, 15]. Adapting to directional motion in sectors having an angle of 90° would constrain the axis of the mean directions of the phantom MAE to be nearly orthogonal to the axis of the mean directions of adaptation [13, 15], which should prevent the involvement of low-level motion detectors (e.g., V1 motion detectors). Albright [31] investigated direction selectivity of MT neurons with three types of moving stimuli: oriented slits, single dots, and random-dot fields. All of the recorded MT neurons (n = 110) were directionally selective and exhibited a broad range of direction-tuning bandwidths to all stimuli ranging from 32 to 186 deg (average: 95 deg). However, in V1 neurons, compared with MT neurons, direction-tuning bandwidths were narrower (average: 68 deg), moreover, V1 responses to moving stimuli were weaker, and bidirectional tuning was more common. In addition, the mean orientation-tuning bandwidth in V1 was also significantly narrower than that in MT (on average 64 deg vs. 52 deg, for MT and V1 neurons respectively). Since motion selectivity in V1 reflects relatively narrow orientation and direction-tuning bandwidths, V1 cells can hardly code for motion signals in the test phase that are orthogonal relative to those of the adapting phase [32].

There is psychophysical evidence that MAE originating from adaptation to complex motion (e.g., spiral motion) is reduced when using continuous flash suppression during adaptation (CFS [33]). The CFS is a variant of binocular rivalry (i.e., when different images are presented to each eye, inducing a rival percept that alternates between the two images), in which the dominant eye is presented with a dynamic mask, whereas the non-dominant eye is presented the adapting pattern. On the other hand, differently from the binocular rivalry, the adapting pattern is suppressed and remains undetectable even after an exposure of several seconds [33–35]. Using drifting gratings Maruya et al. [34] tested the MAE seen in stationary (static MAE or sMAE) and dynamic test patterns (i.e., counterphase flickering; dynamic MAE or dMAE) under CFS. The results showed that in the same-eye condition (i.e., when adapting and test patterns were presented to same eye) sMAE and dMAE occurred both when the adapter was visible and when it was suppressed, though the relative magnitude of the aftereffect was reduced with invisible adapters. However, in the different-eye condition (i.e., when adapting and test patterns were presented in different eyes), stationary test patterns did not produce any MAE, neither with visible nor invisible/suppressed adapters. On the other hand, when using dynamic test patterns robust inter-ocular transfer (IOT) was observed in the visible condition, but the IOT component of dMAE disappeared when the adapter was suppressed. These results suggest that low-level adaptation survives under total binocular suppression, and the disappearance of IOT in the dMAE suggests that high-level (binocular) motion detectors do not adapt when the adapting pattern is suppressed from awareness.

In the present study we investigated the phantom MAE using a crowding paradigm. In crowding, the discrimination of a target stimulus presented in the periphery of the visual field is impaired by the presence of nearby stimuli or flankers [36, 37]. Moreover, when a target stimulus is surrounded by flankers it cannot be processed consciously, i.e., observers are not aware of specific features of a crowded target like, for example, its orientation or motion direction [32, 38–43].

There are several explanations for crowding. For example, crowding has been attributed to the integration of visual features over an area defined approximately as half of the target eccentricity [44, 45], which is set pre-attentively [37]. According to this bottom-up hypothesis, crowding occurs when target and distracters’ features are integrated within the same receptive field [45]. This is because in the periphery of the retina receptive fields are much larger than in the fovea and several features from adjacent stimuli are integrated into the same receptive field [37, 44, 45]. Despite the fact that crowding occurs mainly in peripheral vision there is psychophysical evidence that it also occurs in foveal vision [46, 47]. Thus, it is possible that when two or more visual items are within the same integration area their features are combined (or exchanged) resulting in degraded perception [37, 39, 48]. Therefore, based on Pelli et al. [37], crowding occurs at an intermediate level at which the output of single feature detectors is integrated within what they called “integration fields”.

However, there are also top-down explanations for crowding. These theories state that crowding could depend on coarse spatial resolution of attention in the peripheral visual field [39, 49, 50] or to unfocussed attention [51]. Yeshurun and Rashal [52] found that precueing the target location diminishes the effects of crowding and reduces the critical distance for crowding (i.e., the target-to-flankers distance at which the flankers no longer interfere with target identification). Other accounts of crowding focus on the increased spatial uncertainty in the periphery of the visual field [53]. In this case the information integration between target and distracters could arise from a loss of spatial position information or source confusion [48, 54–56]. Dakin et al. [54] used an orientation-averaging task, in which observers judged the mean orientation of a set of oriented elements either in isolation or crowded by other elements with random orientation. Observers performed also a concomitant attentional task. The results showed that crowding increases the local uncertainty of the orientation of single elements, thus limiting the estimation of local orientation, while distraction reduced the overall global efficiency, i.e., the orientation information was pooled over a smaller number of elements. Furthermore, neuroimaging and psychophysical studies have shown that crowding is a multistage process [49, 57–61] involving low- and high-levels of visual analysis, thus representing an important tool to investigate the role of awareness at the global motion level where optic flow components are processed. There is psychophysical evidence that high-order MAEs from adaptation to complex motion (e.g., spiral patterns [38]), second-order motion [62] and apparent motion [42] persist, though reduced in strength, despite a lack of awareness of the direction of the adapting stimulus. However, to date the phantom MAE has been scarcely investigated and there are no data about the role of awareness in building up such an aftereffect. In this study using clockwise and counterclockwise globally moving dots we measured the phantom MAE in both crowded and non-crowded conditions to assess whether the phantom MAE is evident when observers are not aware of the adapting direction.

Additionally, it is well established that extrastriate areas are strongly modulated by attention. Cell recordings from the macaque area MT [63–65] and neuroimaging data from MT and MST in humans [66–69] have revealed attentional modulation of motion processing.

However, in the case of the MAE the results are contradictory; while some studies reported that distraction affects the strength of the MAE [32, 33, 70–73], other studies reported no effect of attention on motion adaptation [74–77]. For example, Morgan [76] adapting to expanding motion did not report an effect of attention on any of the measures of adaptation adopted (i.e., duration and a speed nulling), suggesting that the methods used to show the effect of distraction (e.g., the duration of the aftereffect) could be potentially susceptible to bias. Thus, we also assessed the role of attention in the phantom MAE with crowded and non-crowded adapting patterns. In particular, we diverted attention from the adapting stimulus using a highly attention-demanding rapid serial visual presentation (RSVP) task.

The aim was to investigate whether the phantom MAE needs either attention or awareness, or both to be established. Moreover, manipulating awareness and attention independently allowed us to test for the interaction of these two variables at high-levels of motion processing.

Based on previous findings we hypothesized that both conventional and phantom MAEs would survive crowding, though they should be reduced in magnitude [38, 40, 42, 62], with the phantom MAE being weaker than the conventional MAE [13–15]. However, when diverting the attention from the adapter we may observe different outcomes: (i) in the *non-crowded* condition distraction could potentially affect the magnitude of both conventional and phantom MAEs [32, 33, 70–73]. On the other hand, if attention does not affect MAE as suggested by Wohlgemuth [74] and Morgan [75–77], we should expect similar results to those observed in the non-crowded and non-distracted condition (i.e., baseline condition), (ii) in the *crowded* condition distraction may completely suppress both the conventional and phantom MAEs, suggesting that visual motion adaptation depends on both awareness and attention [33]. This is because if crowding results from limited attentional resources its effect should increase while attention is engaged in a secondary task (RSVP), and thus less available [54]. Alternatively, no effect of attention in the crowded condition would provide not only further support for independence of MAE and attention [74–77], but it would also suggest a dissociation between awareness and attention; that is, crowding would affect both conventional and phantom MAEs regardless the presence of a distracting task [78].

## METHODS: EXPERIMENTS 1 AND 2

### Participants

Eleven participants took part in the attention non-distracted experiment and in the attention distracted experiment. One of the authors, our student assistant and three naïve participants took part in both experiments. All participants had normal or corrected to normal visual acuity. This study was approved by the Ethics Committee of the University of Regensburg (http://ethikkommission.uni-regensburg.de/). Written informed consent was obtained from each participant prior to the enrolment in the study.

### Apparatus

Stimuli were displayed on a 22” LCD DELL P2210 monitor with a refresh rate of 60 Hz. We generated the stimuli with Matlab Psychtoolbox [79, 80]. The screen resolution was 1024 × 768 pixels with an aspect ratio of 4:3. Each pixel subtended 2.2 arcmin. The minimum and maximum luminance of the screen were 0.18 and 211.2 cd/m^2^, respectively, and the mean luminance was 108.4 cd/m^2^. Luminance was measured with a CRS Optical photometer (OP200-E). A gamma-corrected lookup table (LUT) was used so that luminance was a linear function of the digital representation of the image. To monitor central fixation the gaze position of the right eye of the participants was continuously measured using a CRS High-Speed Video Eye-Tracker (Cambridge Research System Ltd, Rochester, Kent, UK; average spatial resolution: 0.125 to 0.25 deg of visual angle, sampling rate: 250 Hz).

### Stimuli

Stimuli consisted of globally moving dots with 100% coherence, and presented in two circular sectors subtending a central angle of 90° [13, 16]. Each moving patch was composed of 50 dots (i.e., 25 dots per sector): 50% of the dots were white (211.2 cd/m^2^) and 50% black (0.18 cd/m^2^) with a diameter of 0.12 deg. Dots moved on a gray background of the same mean luminance of the screen (108.4 cd/m^2^). The whole circular array was displayed within a Gaussian envelope with a contrast of 0.99 and σ = 1.9 deg, thus subtending a circular aperture of 3 deg diameter and a density of 14 dots/deg^2^ (Fig. 1). It should be noted that Snowden and Milne [15] used a similar circular aperture of diameter 5 deg. In addition, moving stimuli with blurred edges are likely to reduce retinotopic effects and ensure that motion adaptation occurs at high level of motion processing [81, 82]. The motion sequence was calculated offline and stored in three-dimensional (*x*, *y*, *t*) Matlab matrices; *x*, *y* represent the *x* and *y* position of each dot at each display frame, and *t* the frames of the motion sequence. Matrices in turn were stored in the secondary computer memory. On the first frame dots were randomly positioned within the circular sectors and were displaced by 0.15 deg on each subsequent frame producing an angular speed of 6.06 rad/s and corresponding to a tangential speed of 9.1 deg/s (angular speed was calculated as *v*/*r*, where *v* is the tangential speed and *r* is the radius). Dots were assigned a direction according the type of motion being simulated (i.e., clockwise or counterclockwise) [5, 26] (see Fig. 1). It should be noted that the clockwise or counter-clockwise motion was defined by dots moving within each stationary circular sector and not by the motion of the circular sectors themselves.

In contrast to that used by Snowden and Milne [15, 26] and Graziano et al. [1], the local speed of each dot did not vary with distance from the origin, thus the circular motion did not correspond exactly to rotation of a rigid body. However, Morrone et al. [8] found no differences between the blood-oxygen-level-dependent (BOLD) responses to rigidly rotating patterns (where local speed varied with radius) and those with constant local speed [8, 13].

Local motion signals were strongly minimized by implementing a limited lifetime; that is, after 50 ms each dot vanished and was replaced by a new dot of the same color at a different randomly selected position within the same sector [15, 26, 83, 84]. Thus, dots appeared and disappeared asynchronously on the display. In addition, moving dots that traveled outside the sector were also replaced by a new dot at a different random location within the same sector, thus always maintaining a constant density. Test stimuli were dynamic noise patterns (0% coherence) in which dots moved linearly in a wide range of directions [85], but had the same temporal characteristics of the dots used in the adapting stimuli. Test stimuli were also arranged in two circular sectors subtending a central angle of 90° [13, 15]. Each noise dot had a randomly selected initial direction of displacement and then continues to move linearly in the same direction on successive frames for the duration of its lifetime. This type of noise has been labeled by Scase et al. [86] as “random-direction” noise. After the initial adaptation period we presented the test pattern, the sectors of which could overlap with those of the adapting stimulus (conventional MAE), or could be displayed in the non—adapted sectors (phantom MAE) (Fig. 1).

### Procedure

Participants sat in a dark room at a distance of 57 cm from the screen. The participant's head was stabilized by asking him/her to rest his/her chin on a chinrest. Viewing was binocular. Participants were instructed to fixate at the center of the screen. Fixation stability was tracked during the experiments.

### Attention non-distracted experiment

**Fig 2:**
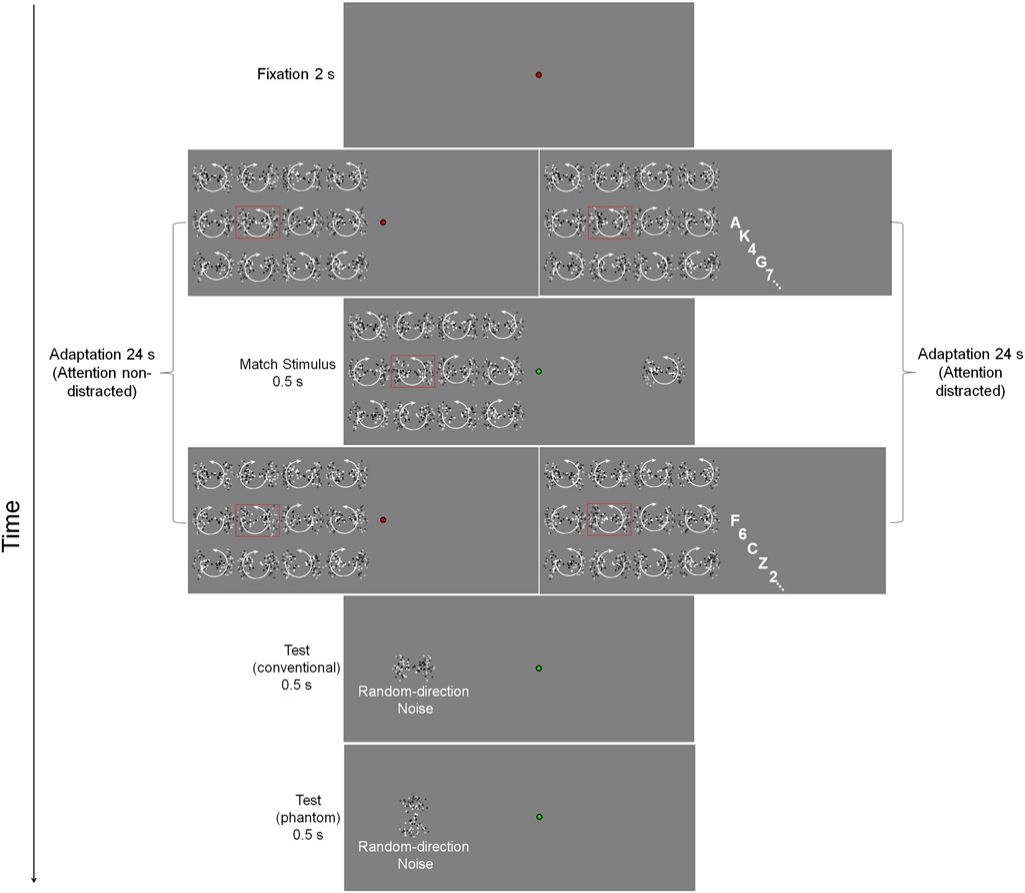
Graphical sketch of the procedure. An initial fixation point of 2 s is followed by 24 s of rotational motion adaptation. The adapting stimulus (indicated by the red frame) moves clockwise and it is crowded by moving flankers. At a certain time-point, chosen randomly between 2 and 22 s during the adapting period, a match stimulus is presented for 0.5 s in the opposite visual hemi-field. After the adaptation period, a test pattern is presented. The sectors of the test pattern can either overlap (conventional MAE) or be orthogonal (phantom MAE) relative to those of the adapting stimulus. The figure shows also adaptation with the central RSVP task (Experiment 2; attention distracted). We also employed a non-crowded condition (not shown) in which flankers were stationary. See text for more details about the procedure.

In the first experiment participants were adapted to a rotating moving adapter in a crowded or in a non-crowded condition. In the crowded condition the adapter was flanked by similarly moving patches and participants were adapted to the adapter’s motion while not being aware of its motion direction. In the crowded condition moving patches were arranged in a 3 × 4 matrix, with the adapting stimulus located in the 2^nd^ row and 3^rd^ column when the whole configuration was displayed in the right visual hemi-field and in the 2^nd^ row and 2^nd^ column of the matrix when the configuration was displayed in the left visual hemi-field (see Fig. 2). Thus the center of the adapting stimulus presented in the left or right visual hemi-field had always the same distance from the central fixation point. In the matrix the center-to-center distance between moving patches was 3.2 deg, whereas the distance from the center of the moving adapter to the fixation point was 9 deg (Fig. 2). Therefore, the eccentricity of the center of the adapting stimulus was 9 deg and it was the same for all the observers. Only the motion direction of the adapting stimulus was randomized and counterbalanced across trials, whereas the direction of the (eleven) flankers was randomized on a trial basis. However, on every trial, 50% of the patches moved clockwise and 50% moved counter-clockwise. Such distribution of directions within the adapting matrix was adopted to avoid global (or remote) adaptation from patterns surrounding the adapter stimulus, since directions average to zero [62]. In addition, participants were always instructed with respect to the location of the moving adapter before the experiment started.

Each session started with a calibration of the eye-tracker, subsequently participants heard a 500 Hz tone of 50 ms duration. The adaptation sequence started once the gaze position of the participants remained inside a critical square window (area: 2.25 deg^2^) for 2 s. The fixation window surrounded a bull’s eye-fixation point (red center [diameter: 0.26 deg] and black surround [diameter: 0.42 deg]). The observers had to maintain their gaze on the central fixation point and were adapted for 24 s to the moving patterns. Stimuli in the matrix had always the same orientation, i.e., the sectors were displayed along the vertical or horizontal axis (Fig. 2).

At a certain time-point during the adapting period, chosen randomly between 2 and 22 s, we presented a match stimulus for 500 ms in the opposite visual hemi-field of the adapting stimulus but at the same eccentricity with respect to the fixation point, so that the match stimulus resulted in the mirrored location relative to the adapting stimulus. When the match stimulus appeared the central part of the fixation point turned to green for 2 s. During this 2-s interval observers had to judge whether the match and the adapting stimuli were moving in the same or different direction (i.e., two-alternative forced-choice; 2AFC) by pressing one of two designated keys on a standard computer keyboard. The configuration of the match stimulus was the same as that of the adapting stimulus but could have had the same or different direction of rotation. A match stimulus was employed in order to assess whether the observers were aware of the direction of the adapter during the adapting period. While the match stimulus was presented, the moving patches in the matrix (i.e., adapter and flankers) continued to move. As soon as the observers performed the 2AFC task, the center of the fixation point turned again to red until the end of the adapting period. Immediately after the adapting phase, the center of the fixation point turned to green, flankers were removed and at the same spatial location of the adapting stimulus we presented a dynamic test pattern (i.e., noise), the sectors of which could overlap with those of the adapting stimulus (conventional MAE condition) or could be displayed in the non—adapted sectors (phantom MAE condition) (Fig. 2). Observers judged whether the motion direction of the test pattern was clockwise or counter-clockwise (Method of Single Stimuli; MSS [87]). The keys were the same to those used for the above described 2AFC task (i.e., the key “M” was used to indicate same direction and clockwise motion, and the key “Z” for different direction and counter-clockwise motion). Afterwards the central part of the fixation point turned to blue and this signaled the start of the inter-trial interval (5 s). Thus, on each trial participants had to perform two tasks: (i) they were asked to judge whether a match stimulus presented at a random time-point during the adapting phase had the same or different motion direction with respect to the adapting pattern (2AFC task), and (ii) immediately after the adapting phase they had to judge whether a (noisy) dynamic test pattern was moving either clockwise or counter-clockwise (MSS). It should be noted that we employed two different tasks to avoid response bias on the test pattern arising from the previous judgment of the match stimulus during adaptation.

The non-crowded condition was the same as the crowded condition with the exception that the flankers were stationary across the entire adapting period.

We conducted 16 sessions: 8 sessions for the crowded conditions and 8 sessions for the non-crowded condition. Crowded and non-crowded sessions were presented block-wise. The presentation order of the sessions was randomized across participants. Each session consisted of 8 trials: 2 adapting directions (clockwise and counter-clockwise) x 2 test stimulus conditions (conventional and phantom) x 2 visual hemi-fields (left and right visual hemi-fields). Overall each participant performed 128 trials. The (x, y) position of the right eye was measured during each trial with the eye-tracker. During the inter-trial interval (5 s) we suspended eye-position recording. Data were analyzed offline and trials in which fixation strayed from the critical window surrounding the fixation point were discarded from the analysis.

### Attention distracted experiment

Experiment 2 was similar to Experiment 1, except for the addition of an attentional task at fixation (Fig. 2). The attentional task was similar to that employed by Kaunitz et al. [33] and consisted of a rapid serial visual presentation (RSVP) of letters and digits. In particular, we used all the letters of the alphabet and digits from 1 to 4 and from 6 to 9. Letters and digits were presented in Arial font and subtended 0.6 deg. Each session started with a calibration of the eye-tracker, subsequently participants heard a 500 Hz tone of 50 ms duration. The adaptation sequence and the letter-digit stream started once the gaze position of the participants remained inside a critical (virtual) square window for 2 s. Letters and digits appeared for 300 ms interleaved with 200 ms with a blank (presentation rate: 2 Hz). Letters and digits were displayed at the center of the screen and replaced the fixation point during the adapting period. At a certain time-point during the adapting period, chosen randomly between 2 and 22 s, we presented a match stimulus for 500 ms in the opposite visual hemi-field of the adapting stimulus but at the same eccentricity with respect to the fixation point (i.e., 9 deg). When the match stimulus appeared the letters/digits stream was stopped and a green fixation point was displayed. The interruption of the stream lasted for 2 s, to allow the observers to compare the motion direction of the adapting stimulus to that of the match stimulus (Fig. 2). Observers were instructed to consider digits as the critical stimuli and letters as distracters and to respond as fast as possible whether the digits were above or below “5”. The keyboard keys were “M” to indicate digits > 5 and “Z” to indicate digits < 5. Letters and digits were randomly chosen but with the constraint that two identical letters or digits could not be presented consecutively. To ensure high attentional load we used a rate of 1/3 of digit presentation (i.e., digits among letters presented) [33]. Similarly to Experiment 1, while the match stimulus was presented, the moving patches in the matrix (i.e., adapter and flankers) continued to move. As soon as the observers responded to the match stimulus the green fixation point disappeared and the letters/digits stream started again until the end of the adapting period. Immediately after the adapting phase another green fixation point was displayed, flankers were removed and at the same spatial location of the adapting stimulus a dynamic noisy test pattern was displayed, the sectors of which could overlap with those of the adapting stimulus (conventional MAE condition) or could be displayed in the non—adapted sectors (phantom MAE condition) (Fig. 2). Observers judged whether the motion direction of the test pattern was clockwise or counter-clockwise (MSS). The keys were the same to those used in Experiment 1.

## RESULTS: EXPERIMENTS 1 AND 2

### Eye-Movements

Trials in which the subjects’ eyes moved outside the critical spatial window were discarded from the analysis. However, this rarely occurred during the adapting period (on average <3% of all trials) and it never happened during the test phase. In particular, for the attention non-distracted experiment we found 2.8% (SEM: 0.7%) and 2.1% (SEM: 0.7%) of trials with excessive eye movements in the non-crowded and crowded conditions, respectively. For the attention-distracted experiment we found 1.4% (SEM: 1%) and 1.3% (SEM: 0.6%) of trials with excessive eye movements in the non-crowded and crowded conditions, respectively. A Lilliefors test performed on the percentage of fixational eye movements separately for the two experiments revealed a violation of normality assumption (*p* = 0.03 and *p* = 0.0001, respectively). We then used the non-parametric Kruskal-Wallis test separately for the two experiments to test for an effect of the adapting condition (i.e., non-crowded vs. crowded). In the case of the attention non-distracted experiment the Kruskal-Wallis test did not determine any significant difference between the fixational eye movements in the non-crowded and crowded conditions (*χ^2^* = 1.02, *df* = 1, *p* = 0.31). The same result was obtained in the case of the attention-distracted experiment (*χ^2^* = 0.49, *df* = 1, *p* = 0.48).

In order to compare the percentage of fixational eye movements between the two experiments (i.e., attention not distracted vs. attention distracted), we pooled the percentage of eye-movements obtained in the two adapting conditions (i.e., non-crowded and crowded) relative to each experiment. Since an Ansari-Bradley test confirmed that the distribution of eye-movements in the attention non-distracted experiment and the distribution of eye movement in the attention-distracted experiment have the same variance (*W* = 68, *p* = 0.84), we performed a Wilcoxon two-sample test. The results showed no significant difference between the fixational eye movements between the two experiments (*Signed Rank* = 41, *p* = 0.19).

### 2AFC task during motion adaptation

**Fig 3:**
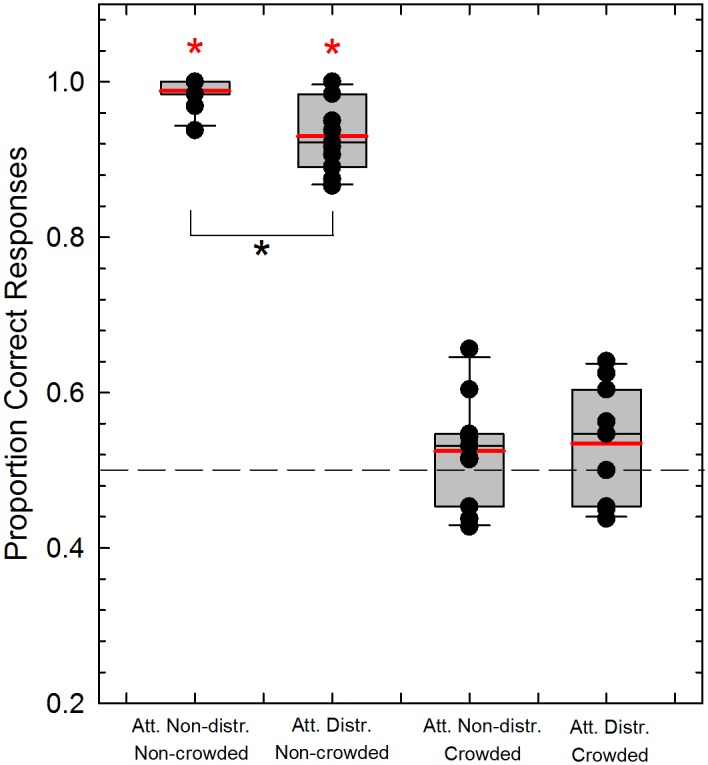
Mean proportion of correct responses for the 2AFC task in Experiments 1 (N = 11) and 2 (N = 11). Box plots illustrate the mean proportion of correct responses for the 2AFC task during the adapting phase for the attention non-distracted and the attention-distracted experiments, and for the crowded and non-crowded adapting conditions. Observers had to report whether the match stimulus was moving in the same or different direction with respect to the adapting stimulus. The black and red continuous lines inside the boxes represent the median and the mean respectively for each condition. Black dots represent the raw data for each participant in each condition. Whiskers represent the minimum and maximum proportion of correct response for each group. The height of each box defines the interquartile range (IQR) (i.e., Q_3_-Q_1_). The red asterisks represent a significant difference with respect to the chance level (dashed line), whereas the black asterisk represents a significant difference between the attention non-distracted and the attention distracted experiments for the non-crowded condition (see text for more details).

Fig. 3 shows the box plot of the proportions of correct responses obtained in the 2AFC task performed during the motion adaptation phase. A Lilliefors test performed on the proportion of correct responses separately for the attention non-distracted and the attention distracted experiments reported a violation of normality assumption (*p* = 0.0001 and *p* = 0.002, respectively), motivating us to use non-parametric statistics.

A Kruskal-Wallis test was used separately for the two experiments to test for an effect of the adapting condition (i.e., non-crowded vs. crowded). For the attention non-distracted experiment the Kruskal-Wallis test revealed a significant difference between the non-crowded and crowded conditions (*χ^2^* = 16.32, *df* = 1, *p* = 0.0001). In addition, we also performed a Bonferroni-corrected one-sample Wilcoxon signed rank test with respect to a median of 0.5 (critical *p* = 0.025). The Wilcoxon test indicated a significant difference in the case of the non-crowded condition (*Signed Rank* = 66, *p* = 0.0001), but not a significant difference for the crowded condition (*Signed Rank* = 43, *p* = 0.41) (Fig. 3).

The same analysis was conducted for the attention-distracted experiment. A Kruskal-Wallis test revealed a significant difference between the non-crowded and crowded conditions (*χ^2^* = 15.81, *df* = 1, *p* = 0.0001). A Bonferroni-corrected one-sample Wilcoxon signed rank test with respect to a median of 0.5 (critical *p* = 0.025) revealed a significant difference for the non-crowded condition (*Signed Rank* = 66, *p* = 0.0001), but no significant difference for the crowded condition (*Signed Rank* = 35.5, *p* = 0.14).

An Ansari-Bradley test revealed that, for the non-crowded condition, the proportion of correct responses obtained in the attention non-distracted and in the attention distracted experiments have the same variance (*W* = 69.5, *p* = 0.63), the same was obtained in the case of the crowded condition (*W* = 73.5, *p* = 0.34). A Wilcoxon two-sample test between the proportion of correct responses obtained in the attention non-distracted and in the attention distracted experiments for the non-crowded condition revealed a significant difference between the accuracy obtained in the two experiments (*Signed Rank* = 54, *p* = 0.004) (Fig. 3). On the other hand, the Wilcoxon test did not reveal any significant difference between the accuracies obtained in the attention non-distracted and attention distracted experiments, for the crowded condition (*Signed Rank* = 31, *p* = 0.88) (Fig. 3). We conclude from these results that crowding indeed blocked subjects’ awareness with respect to the direction of the adaptation pattern. Distracting attention away from the adaptation pattern with the central RSVP task only had a moderate effect in the non-crowded condition and no effect in the crowded condition.

### Conventional and Phantom MAEs

**Fig 4:**
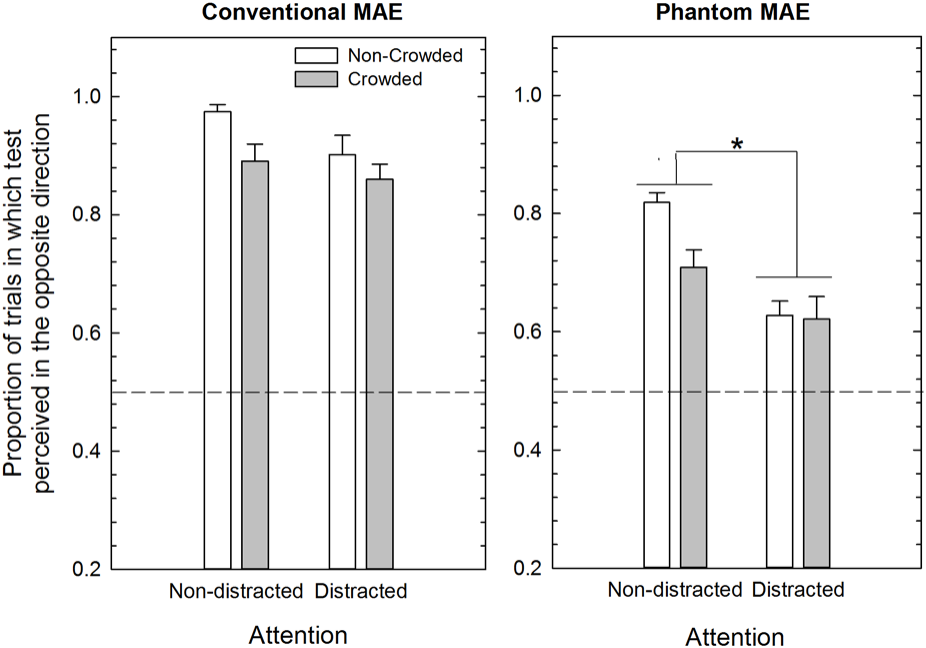
Mean proportion of MAE in Experiments 1 (N = 11) and 2 (N = 11). Mean proportion of trials in which observers judged the test stimulus as drifting in the opposite direction with respect to the adaptation stimulus is shown for the conventional MAE and phantom MAE. The difference between adjacent bars shows the amount of conventional and phantom MAE reduction in the non-crowded vs. crowded conditions as a function of the attentional condition. The asterisk in the right panel indicates a significant difference between the attention non-distracted and the attention distracted experiments in the case of the phantom MAE (see text for more details). Error bars ± SEM.

Fig. 4 shows the results obtained for the conventional and phantom MAE in crowded and non-crowded conditions when attention was not distracted and when it was distracted from the moving adapter. Data of the two adapting motion directions (i.e., clockwise and counter-clockwise) were pooled to increase statistical power. A Lilliefors test conducted separately for the attention non-distracted and the attention-distracted conditions revealed that in the two conditions data were normally distributed (*p* = 0.20 and *p* = 0.15, respectively). In addition, since Mauchly’s test for sphericity was significant for both experiments (*p* = 0.008 and *p* = 0.03), we applied the Greenhouse-Geisser’s correction for the degrees of freedom. A mixed-model ANOVA including as within-subjects factors test condition (conventional vs. phantom), adapting condition (non-crowded vs. crowded), and attention as between-subjects factors, showed a significant effect of attention (*F*(1,20) = 10.70, *p* = 0.004, *partial-η* ^2^ = 0.35), a significant effect of the test condition (conventional vs. phantom) (*F*(1,20) = 157.93, *p* = 0.0001, *partial-η* ^2^ = 0.89), a significant effect of the adapting condition (crowding vs. no crowding; *F*(1,20) = 17.84, *p* = 0.0001, *partial-η* ^2^ = 0.47), a significant interaction between attention and test condition (*F*(1,20) = 6.68, *p* = 0.018, *partial-η* ^2^ = 0.25) and a significant interaction between attention and adapting condition (*F*(1,20) = 6.54, *p* = 0.019, *partial-η* ^2^ = 0.25).

With respect to the interaction between attention and test condition (i.e., conventional and phantom MAEs) post-hoc Bonferroni corrected pairwise comparisons showed a significant difference for the phantom MAE (Fig. 4, right panel) in the condition where attention was not distracted from the adapter compared to when observers had to perform the central RSVP task (*p* = 0.001). On average, the phantom MAE is reduced by 14% (SEM: 3.5%) when the participants’ attention is distracted compared to when it is not distracted during adaptation. On the other hand, for the conventional MAE (i.e., when adapting and test sectors spatially overlapped) distracting attention with the RSVP task during adaptation only reduced the strength of the MAE by 5.2% (SEM: 3.3%) relative to the attention non-distracted condition (*p* = 0.13).

Post-hoc Bonferroni corrected pairwise comparisons were also performed for the interaction between attention and adapting condition (i.e., non-crowded vs. crowded). The results suggest that the attention-distracted condition was more effective for the non-crowded condition (*p* = 0.0001) than for the crowded condition (*p* = 0.12). In particular, in the case of the phantom MAE we obtained a reduction of 19% in the non-crowded condition when the attention was distracted from the adapting stimulus (Fig. 4, right panel). Overall, the results indicate that attention had a significant effect on the phantom MAE, but no effect on the conventional MAE. Moreover, attention was more effective for the non-crowded condition than for the crowded condition.

We also performed a series of one-sample Bonferroni corrected t-tests (critical *p* = 0.0125), separately for each experiment, relative to chance level to assess whether the MAEs obtained across the different stimulus conditions were significantly above chance. All the comparisons indicated that the observed results were significantly above chance (*p* < 0.0125).

## DISCUSSION: EXPERIMENTS 1 AND 2

The results suggest that distracting attention mainly affects the strength of the phantom MAE when the adapter was not crowded. That is, a greater difference was found for the phantom MAE in the non-crowded condition between the attention distracted and non-distracted conditions (i.e., 19% difference) (Fig. 4, right panel). However, interpretation of the effect of attention on the conventional and phantom MAE is not straightforward, because the phantom MAE in the non-crowded and non-distracted condition (i.e., baseline condition for the phantom MAE) is much weaker than the conventional MAE seen in the same condition (~16% difference). Thus, it is possible that the effect of distraction may be apparent only for the weaker adapter in the case of the phantom MAE [13, 15]. Indeed, it should be noted that for the conventional MAE in the non-crowded and non-distracted condition there is a ceiling effect; that is, the conventional MAE was seen in ~97% of trials. Therefore the main effect of attention and the interaction between attention and test condition (i.e., conventional vs. phantom) may depend only on the phantom MAE, for which the effects of attention were more evident. Thus we hypothesized that in the case of the conventional MAE the absence of a significant effect of distraction on the non-crowded condition may be due to such a ceiling effect [88]. In order to address this issue we performed an additional experiment in which we attempted to match the strength of the conventional MAE (in both the non-crowded and crowded conditions) to that of the phantom MAE in the non-distracted condition. This was done in order to obtain comparable conventional and phantom MAEs in the attention non-distracted condition and in non-crowded and crowded conditions.

Additionally, another interesting result of Experiments 1 and 2 is the lack of an effect of distraction in the crowded condition. Although the conventional MAE is much stronger than the phantom MAE in the crowded condition, there is no evidence for an effect of attention. These results suggest that for high-level motion processing crowding is not modulated by attention, indicating a possible dissociation between these two mechanisms [78].

## METHODS: EXPERIMENT 3

### Participants

One author (AP) and ten naïve participants took part in the experiment. All participants had normal or corrected to normal visual acuity. All participants took part voluntarily, and all received compensation (except for the author) for their time. In addition, all participants gave written informed consent prior to their inclusion in the experiment.

### Stimuli and Procedure

Stimuli were the same as used in the previous experiments. The procedure was the same as reported for the previous experiments except that: (i) we used a within-subjects design, i.e., the same observers took part in both the attention non-distracted and the attention distracted conditions, (ii) we varied the motion coherence of the adapter of the conventional MAE, in both the non-crowded and crowded conditions. This was done only for the non-distracted condition in order to match the relative strength of the conventional MAE with respect to that of the phantom MAE, and (iii) the 2AFC task during the adapting phase was now performed at the end of each trial. After the observers judged the motion direction of the test stimulus (i.e., either clockwise or counter-clockwise; MSS task), they were asked to judge whether a match stimulus was moving in the same or different direction to that of the adapting pattern (2AFC task). This was done to avoid any interruption of the RSVP task during the adapting phase in the attention-distracted condition. Indeed, any interruption of the RSVP task may cause a temporary reallocation of the attention during the 500 ms presentation of the match stimulus and the time required to make the response, thus affecting the distraction from the adapting pattern.

### Motion coherence threshold

In Experiment 3 we manipulated, individually for each observer, the motion coherence of the adapting pattern in the non-crowded and crowded conditions of the conventional MAE, in order to match its strength relative to that of the phantom MAE. As in the first two experiments, we used 100% motion coherence during adaptation for all phantom-MAE conditions. Motion coherence thresholds were estimated only in the attention non-distracted condition.

Each session started with a calibration of the eye-tracker, subsequently participants heard a 500 Hz tone of 50 ms duration. The observers were adapted for 24 s to the moving patterns. Immediately after the adapting phase, the center of the fixation point turned to green, flankers were removed and at the same spatial location of the adapting pattern we presented a dynamic test pattern (i.e., noise; duration: 500 ms), the sectors of which always overlapped with those of the adapter (i.e., conventional MAE). Observers judged whether the motion direction of the test pattern was clockwise or counter-clockwise (MSS [87]). Afterwards the central part of the fixation point turned to blue and this signaled the start of the inter-trial interval (5 s). The motion coherence of the adapting pattern was varied using the Method of Constant Stimuli (MCS). On each trial, the motion coherence of the adapting pattern could be one value chosen from an interval ranging from 0% to 100% in steps of 20%, corresponding to 0, 8, 16, 24, 32 or 40 coherently moving dots. It should be noted that the maximum number of coherently moving dots in each circular array was 40, instead of 50 (see the procedure of Experiments 1 and 2). Moreover, it should be noted that in the case of the crowded condition the distracters had always the same motion coherence of the adapting stimulus.

In Experiment 1, where attention was not distracted during adaptation, the phantom MAE was observed in 82% of the trials in the non-crowded condition and in 71% of the trials in the crowded condition. Therefore, we fitted a logistic function [89, 90] to the data to estimate the level of motion coherence that produced a conventional MAE with a 82% likelihood in the non-crowded condition and a conventional MAE of 71% in the crowded condition. Non-crowding and crowding conditions were randomized across participants. Each condition consisted of 8 blocks. Each block consisted of 6 trials. Thus, eight repetitions were performed for each level of motion coherence. Participants either performed first the non-crowded condition (8 blocks) and then the crowded condition (8 blocks) or vice versa.

### Attention non-distracted and attention distracted conditions

The motion coherence thresholds estimated in the previous phase were used in the non-crowding and crowding conditions of the conventional MAE, for the attention non-distracted and the attention distracted conditions. The procedure of the third experiment was identical to that of Experiments 1 and 2, except that the 2AFC task (i.e., when observers judged whether the match stimulus was moving in the same or different direction with respect to the adapting patch) was now performed at the end of each trial. After the response to the test stimulus (MSS task) there was a 2-s delay and then the match stimulus was displayed in the same spatial location to that of the adapting pattern. However, there was not a time-limit to deliver the response for the 2AFC task.

## RESULTS: EXPERIMENT 3

### Eye-Movements

Trials in which the subjects’ eyes moved outside the critical spatial window were discarded from the analysis, which was done for each phase of Experiment 3. In particular, for the motion coherence threshold experiment subjects exhibited excessive eye movements, on average, 4.9% (SEM: 2.1%) and 5.2% (SEM: 2.3%) of trials in the non-crowded and crowded conditions, respectively. A Lilliefors test performed on the percentage of fixational eye movements reported a violation of normality assumption (*p* = 0.0001). Thus, we performed a Kruskal-Wallis analysis to test for an effect of the adapting condition (i.e., non-crowded vs. crowded). The results did not reveal any significant difference between the non-crowded and crowded conditions in terms of percentage of excessive fixational eye movements (*χ^2^* = 0.03, *df* = 1, *p* = 0.86).

In Experiment 3 for the attention non-distracted condition we found 4.7% (SEM: 1.5%) and 2.6% (SEM: 0.9%) of trials where the participants exhibited excessive eye movements in the non-crowded and crowded conditions, respectively. In the attention distracted condition we found 4.1% (SEM: 1%) and 1.8% (SEM: 0.6%) of trials where the participants exhibited excessive eye movements in the non-crowded and crowded conditions, respectively. A Lilliefors test performed on the percentage of trials with excessive fixational eye movements in the two attentional conditions and in the two adapting conditions (i.e., non-crowded and crowded) revealed a violation of normality assumption (*p* = 0.0001). A Friedman test showed no significant differences in terms of percentage of fixational eye-movements across the four conditions tested (*χ^2^* = 5.1, *df* = 3, *p* = 0.16).

### Motion coherence thresholds

The mean percentage of coherently global moving dots required to decrease the strength of the conventional MAE to 82% report probability in the non-crowded condition was 58% motion coherence (corresponding to 23 dots [SEM: 2.7 dots]), whereas the mean percentage of coherently moving dots required to decrease the strength of the conventional MAE to 71% report probability in the crowded condition (thereby matching the report probabilities shown in Fig. 4, right panel, for the non-distracted condition) was 43% motion coherence (corresponding to 17 dots [SEM: 3.2 dots]). A Lilliefors test revealed that the estimated thresholds were normally distributed (*p* = 0.25). A paired-samples t-test revealed a significant difference between the mean coherence thresholds estimated in the non-crowded and crowded conditions (*t*(10) = 1.89, *p* = 0.08).

### 2AFC task

**Fig 5:**
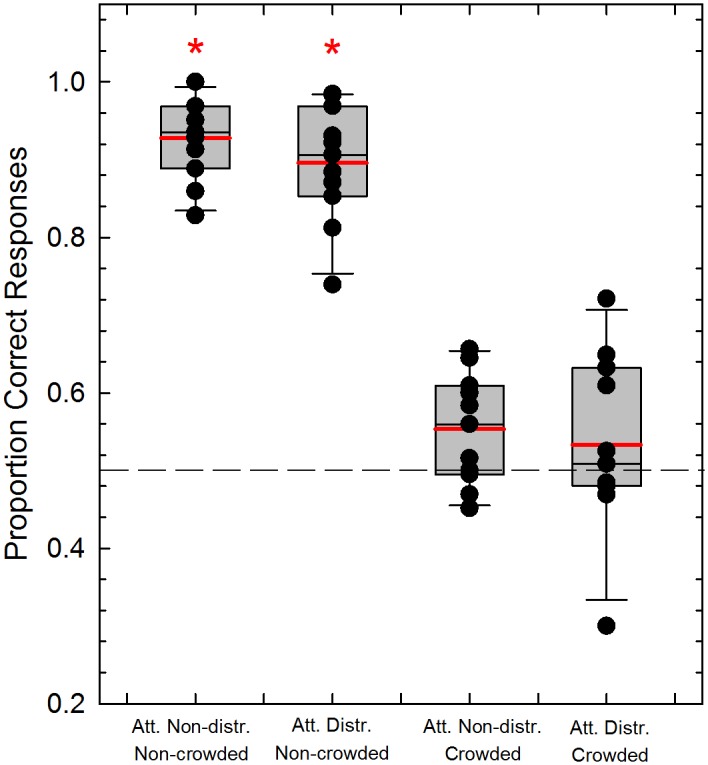
Mean proportion of correct responses for the 2AFC task in Experiment 3 (N = 11). Box plots illustrate the mean proportion of correct responses for the 2AFC task with respect to the perceived direction of the adapting stimulus. The black and red continuous lines in the boxes represent the median and the mean for that condition, respectively. Black dots represent the raw data for each participant and condition. Whiskers represent the minimum and maximum proportion of correct response for each group. The red asterisks represent a significant difference with respect to the chance level (dashed line) (see text for further details).

Fig. 5 shows the box plot of the proportion of correct responses obtained in the 2AFC task performed at the end of each trial when the subjects were asked to report the direction of the adapting pattern. A Lilliefors test performed on the proportion of correct responses, considering the two attentional conditions and the two adapting conditions, revealed a violation of normality assumption (*p* = 0.005). A non-parametric Friedman test including as factors the two attentional conditions and the two adapting conditions (i.e., non-crowded and crowded) revealed a significant difference between these conditions (*χ^2^* = 26.89, *df* = 3, *p* = 0.0001). We performed a series of Bonferroni-corrected Wilcoxon two-sample tests in order to compare the different conditions (critical *p* = 0.025). A Wilcoxon two-sample test conducted between the proportion of correct responses in the attention non-distracted and in the attention distracted conditions for the non-crowded condition did not reveal a significant difference (*Signed Rank* = 47, *p* = 0.24). The same result was found when comparing the proportion of correct responses obtained in the attention non-distracted and in the attention distracted conditions of the crowded condition (*Signed Rank* = 33, *p* = 0.63).

We also performed Bonferroni-corrected one-sample Wilcoxon signed rank test with respect to a median of 0.5 (critical *p* = 0.0125). The Wilcoxon test pointed out a significant difference for the non-crowded conditions: *Signed Rank* = 66, *p* = 0.0001 and *Signed Rank* = 66, *p* = 0.0001, for the attention non-distracted and attention distracted conditions, respectively. However, the Wilcoxon test did not report a significant difference for the crowded conditions: *Signed Rank* = 47, *p* = 0.048 and *Signed Rank* = 40, *p* = 0.56, for the attention non-distracted and attention distracted conditions, respectively.

### Conventional and Phantom MAEs

**Fig 6:**
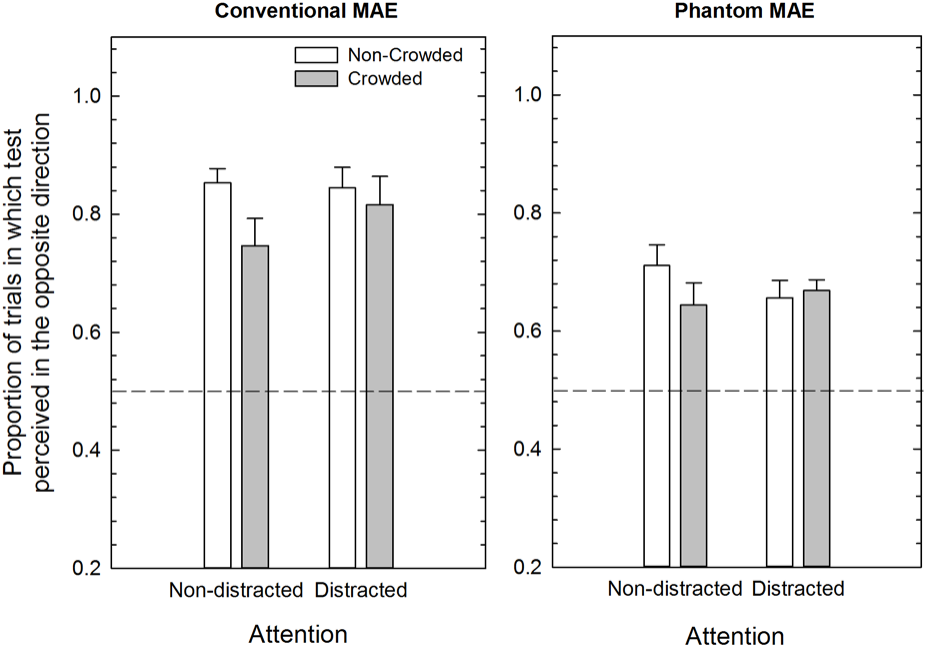
Mean proportion of MAE in Experiment 3 (N = 11). Mean proportion of trials in which observers judged the test stimulus as drifting in the opposite direction with respect to the adaptation stimulus is shown for the conventional MAE and phantom MAE. Error bars ± SEM.

Fig. 6 shows the results obtained in Experiment 3 for the conventional (left panel) and phantom MAEs (right panel) in crowded and non-crowded conditions, when attention was not distracted and when it was distracted from the moving adapter. As done in Experiments 1 and 2 (see above), data of the two adapting motion directions were pooled to increase statistical power.

A Lilliefors test indicated that the data were normally distributed (*p* = 0.45). In addition, since the Mauchly’s test for sphericity yielded a significant value (*p* = 0.018), we applied the Greenhouse-Geisser’s correction for the degrees of freedom. A repeated measures ANOVA including as factors the attention (non-distracted vs. distracted), test condition (conventional vs. phantom), and the adapting condition (non-crowded vs. crowded), showed a significant effect of the test condition (*F*(1,10) = 36.83, *p* = 0.0001, *partial-η* ^2^ = 0.79), a significant effect of the adapting condition (*F*(1,10) = 6.10, *p* = 0.033, *partial-η* ^2^ = 0.38), but no effect of attention (*F*(1,10) = 0.09, *p* = 0.77, *partial-η* ^2^ = 0.009). The ANOVA did not reveal any significant interaction.

In addition, we performed a series of one-sample Bonferroni corrected t-tests (critical *p* = 0.0125), separately for each experiment, relative to chance level to assess whether the MAEs obtained across the different stimulus conditions were significantly above chance. All the comparisons indicated that the observed report probabilities were significantly above chance (*p* < 0.0125).

Since Experiment 3 neither revealed a significant effect of attention nor any significant interaction, we conducted an additional analysis to assess the effect of crowding at high-level of motion adaptation. In particular, we analyzed the proportion of MAE (i.e., proportion of trials in which observers judged the test stimulus as drifting in the opposite direction with respect to the adapter) only for the correct trials in the 2AFC task, for which observers are supposed to be aware of the adapter direction. The purpose of this additional analysis was to assess whether (i) crowding reduces the MAEs because it makes observers unaware of the adapter [91, 92] or (ii) because it degrades the sensory representation of the adapter stimulus affecting the MAE strength, without awareness playing an important role [37, 39, 48]. The first hypothesis predicts that analysing only the correct trials of the 2AFC task the effect of crowding should disappear. On the other hand, the second hypothesis would still predict a significant effect of crowding.

A Lilliefors test indicated that the data were normally distributed (*p* = 0.26). A repeated measures ANOVA including as factors the attention (non-distracted vs. distracted), test condition (conventional vs. phantom), and the adapting condition (non-crowded vs. crowded), showed only a significant effect of the test condition (0.80 [SEM 0.034] vs. 0.69 [SEM 0.022], for conventional and phantom MAEs, respectively) (*F*(1,10) = 14.74, *p* = 0.003, *partial-η* ^2^ = 0.60), but not a main effect of attention (*F*(1,10) = 0.33, *p* = 0.58, *partial-η* ^2^ = 0.03) nor adapting condition (*F*(1,10) = 2.42, *p* = 0.15, *partial-η* ^2^ = 0.19). The ANOVA did not reveal any significant interaction.

## DISCUSSION: EXPERIMENT 3

In Experiment 3 the motion coherence of the adapter relative to the conventional MAE was reduced to match the strength of the phantom MAE (as measured in Experiment 1). However, we still found that the conventional MAE was more pronounced compared to the phantom MAE in both the non-crowded and the crowded conditions. This finding could depend on a certain amount of perceptual learning that took place during the adapting sessions, which either could have shifted the psychometric function towards lower levels of motion coherence or led to increases the steepness of the psychometric function.

Although the repeated-measures ANOVA showed a significant effect of the test condition (conventional vs. phantom), the strength of the conventional MAE we reported in Experiment 3 was reduced with respect to the conventional MAE of Experiment 1. In particular, we found, on average, a 12% reduction of the MAE in the non-crowded condition and a 14% reduction in the crowded conditions, when comparing the phantom to the conventional MAE conditions.

Overall, the results show that when the conventional MAE is not at ceiling there is no main effect of attention nor any significant interaction between attention and test condition (conventional vs. phantom), or between attention and adapting condition (non-crowded vs. crowded). Thus, the main effect of attention and the interactions reported in the previous experiments (i.e., Experiments 1 and 2) could be accounted by the fact that the effect of attention was evident only for the phantom MAE.

In addition, the analysis we conducted on the correct trials of the 2AFC task suggests that at a high level of motion adaptation crowding is likely to suppress awareness of the adapter rather than degrading or altering its sensory representation.

## GENERAL DISCUSSION

In a series of experiments we investigated the role of attention and awareness in modulating high-order motion aftereffects such as the phantom MAE. The adapting and test stimuli were similar to those employed by Snowden and Milne [15] and Pavan et al. [13]. We presented globally and coherently moving dots in two sectors with a central angle of 90° (Fig. 1). To suppress awareness the adapting stimulus was embodied in a matrix of moving flankers that had the same spatiotemporal characteristics but with random clockwise or counter-clockwise directions. We included also a non-crowded condition in which the adapting stimulus was displayed between stationary distracters. Immediately after adaptation to rotational motion in the crowded and non-crowded conditions, we presented a test pattern in the same spatial location to that of the adapting stimulus. The test-stimulus sectors could overlap with those of the adapting pattern (conventional condition) or could be orthogonal to those of the adapting pattern (phantom condition). This procedure was used in separate experiments, where attention was not distracted and when attention was distracted away from the adapting stimulus using a highly demanding central RSVP task.

Across all the experiments we found a weaker MAE in the phantom condition relative to the conventional condition. This result is in agreement with those reported by Snowden and Milne [15] and Price et al. [14], who found weaker phantom MAEs relative to the conventional MAE. It should be noted that this difference is independent of the type of measurement adopted. In the present study we measured the proportion of trials in which the test stimulus was judged as drifting in a direction opposite to that of the adapting pattern, while Snowden and Milne [15] and Price et al. [14] used motion-nulling techniques, wherein they manipulated the coherence and temporal frequency of a dynamic test pattern.

In all the experiments we also found a significant difference between the MAEs obtained in crowded and non-crowded conditions. On average, MAEs in the crowded condition were weaker than those obtained in the non-crowded condition. In Experiments 1 and 2 we found a crowding-dependent reduction of 6% (SEM: 1%), whereas in Experiment 3 we found a reduction of 5% (SEM: 2%). This result is consistent with previous findings, which showed that adapting in crowding condition limits the strength of the MAE [38, 39, 42, 62]. The reason for such differences is still not clear, but as Price et al. [14] pointed out, direction-selective neurons at a high level of motion processing have inhibitory surrounds [93, 94] and there is psychophysical evidence that contextual information as well as relative motion affect the strength of the MAE [91, 92]. Thus, it is possible that the relative motion of the surrounding flankers may partially inhibit the motion signals of the adapter causing a small reduction of the conventional MAE and a much larger reduction of the phantom MAE. On the other hand, crowding may also induce changes in adapter appearance from the pooling of motion signals within motion detectors with large receptive fields, resulting in adapter-flanker averaging that leads to more similarity between adjacent regions of the peripheral visual field [41, 57, 95]. Thus, the effects of crowding might accumulate via adapter-flanker pooling across large receptive fields, which probably exploit inhibitory connections [96]. In order to assess the effect of crowding on the adapter we analyzed the proportion of MAE only for the trials in which observers judged correctly whether the adapter and the match stimulus moved in the same or different direction (i.e., 2AFC task); that is, those trials in which observers should have been aware of the adapter’s direction. The rationale was that if crowding suppresses the awareness of the adapter without degrading its sensory representation the effect of crowding on the resultant MAE should disappear. The results did not reveal a significant effect of the adapter condition (i.e., non-crowded vs. crowded). We speculate that the pooling of motion signals within detectors with large receptive fields may not degrade the representation of the adapting stimulus but could weaken the strength of its motion signal, e.g., because of lateral inhibition [97], thus preventing its conscious access and affecting the strength of the MAE. This finding is also compatible with the hypothesis that crowding depends on compulsory integration of target and flankers over a wide area and possibly within the same receptive field [37, 41, 45, 98], which is probably a pre-attentive mechanisms [37].

The results of Experiments 1 and 2 suggest that distracting attention with the RSVP task affects mainly the phantom MAE seen in the non-crowded condition. However, a comparison between phantom and conventional MAEs might be complicated by a ceiling effect evident for the conventional MAE in the non-crowded condition where subjects attended to the adapter and were aware of its direction. To this purpose Cohen et al. [88] argued that to investigate the relationship between attention and awareness it is necessary that performance on a specific task is below ceiling. In particular, if performance is below ceiling on an attentionally demanding primary task (e.g., visual search), attention is thought to be almost fully engaged by that task [99]. Therefore, if performance with a secondary stimulus is unaffected by the primary task, the secondary stimulus must be consciously perceived without attention. In Experiments 1 and 2 the conventional MAE was at ceiling (97%) in the baseline condition (i.e., non-crowded and non-distracted conditions) and this might have prevented an effect of attention. Thus, we assumed that in Experiments 1 and 2 the main effect of attention and the interaction between attention and type of test stimulus (conventional vs. phantom) may depend only on the phantom MAE, for which the performance is below ceiling [88]. In order to avoid this complication, in Experiment 3 we decreased the relative strength of the conventional MAE by reducing the motion coherence of the adapter. Following this manipulation, we still found an effect of the test condition (i.e., conventional vs. phantom) on the strength of the resultant MAE, but we found no effect of attention [74–77] nor an interaction between attention and the test condition (conventional vs. phantom) or between attention and the adapter condition (non-crowded vs. crowded). It is possible that the outcomes found in Experiments 2 and 3 depend on the different task demands. Experiment 2, for example, requires faster responses to the match stimulus presented during the adapting phase since there was a temporal window of 2 s to respond (see the procedure of Experiments 1 and 2), whereas in Experiment 3 the 2AFC task was performed at the end of each trial, and there was not a time-limit to deliver the response. Additionally, in Experiment 3 bringing the conventional MAE below ceiling might have rendered the task more difficult. However, Experiment 3 was necessary to control for the ceiling effect emerging in Experiments 1 and 2 [88] and to avoid any interruption of the adapting period for the 2AFC task. In general, we propose that further psychophysical experiments are necessary to investigate the role of stimulus configuration and task demands on the interplay between crowding and attention at high level of motion processing.

Overall, it seems that for the stimulus configuration used in the present study attention does not modulate the MAE neither in the non-crowded nor in the crowded conditions. In particular, our results suggest a dissociation between attention and awareness. Crowding exerts an effect on both conventional and phantom MAE even when observers were engaged in the central RSVP task. These findings are in agreement with those reported by Ho and Cheung [78]. In their study observers were adapted to the orientation of a peripheral target crowded by similar elements, and with flankers perceptually suppressed by using CFS [33–35]. Observers also concurrently performed a demanding RSVP task at fixation (i.e., counting the number of red crosses) and had to report the interval in which the test grating appeared after the adaptation period (2IFC). The results showed a crowding effect (i.e., higher contrast detection thresholds) in both the No-CFS and CFS conditions, though there was a stronger crowding effect when flankers were not suppressed than for the condition in which they were suppressed. This is partially consistent with the results of Wallis and Bex [43] who found that crowding could be released when flanking elements at attended locations are suppressed from visual awareness. Most importantly, the results of Ho and Cheung [78] showed that crowding had an effect on the target’s threshold elevation even when observers were required to perform the central RSVP task. These results suggest a dissociation between awareness and attention in crowding. Other studies report that visual experience is likely to depend on the cumulative contribution of awareness and attention that operate independently at the level of the primary visual cortex [100, 101]. Brascamp et al. [100], for example, found that attention and awareness produce response enhancement in phase-sensitive neural channels, present at early stage of visual processing and in phase-insensitive channels, present in higher visual areas. The effects of attention and awareness on phase-insensitive responses were positively correlated, but no correlation was found between the effects of attention and awareness on phase-sensitive responses. These results indicate independence of attention and awareness in early visual areas, and a convergence of their effects at higher levels of visual analysis. Despite the evidence of independence between awareness and attention at early stages of visual analysis, the interplay between awareness and attention at high levels of motion processing needs to be further explored. However, our results report for the first time a dissociation between these two mechanisms in the motion domain.

Other recent studies involving detection of oriented targets [102, 103] and orientation discrimination [104, 105] in crowding conditions pointed out that focused attention plays a crucial role in the mechanism of crowding, modulating its strength [51, 106]. To this purpose, Montaser-Kouhsari and Rajimehr [40] reported weaker adaptation to illusory contours (i.e., two line gratings abutting each other with a phase shift) when attention was diverted from the adapting stimuli in the crowding condition. The authors concluded that attention may subliminally enhance the processing of visual information of the crowded item, when the target and flankers are more finely spaced than the spatial resolution limit of attention [39]. Additionally, Spivey and Sprin [107] showed that selective visual attention modulates the magnitude and presence of the direct tilt after-effect (TAE). In a series of experiments the authors reported that attending to one of two identically oriented gratings, equidistant from fixation, increased the magnitude and occurrence of the TAE for the attended region relative to the unattended region. In addition, they showed that attending to one of two colored gratings in a symmetric plaid pattern produced a direct TAE in the direction opposite the orientation of the attended grating. Taken together these findings suggest the possibility that the tilt after-effect is modulated by attention, whereas the motion after-effect is not [75–77]. Further psychophysical investigations will be necessary to better investigate this possible dissociation with respect to the role of attention at different stages of visual analysis and for different stimulus characteristics (e.g., orientation and directional motion).

Despite the fact that we did not find any attentional modulation of the conventional and phantom MAEs, neither in the non-crowded nor in the crowded condition (Experiment 3), there is recent psychophysical evidence that attention can modulate motion adaptation when awareness is diverted from the adapting stimulus. Kaunitz et al. [33] employed a CFS paradigm [34, 35] to investigate the role of awareness on the spiral MAE, and used a RSVP task to withdraw attention from the adapting pattern. CFS is different from crowding since it suppresses stimuli from awareness by binocular rivalry, displaying to the dominant eye a dynamic noise masker and the adapting pattern in the non-dominant eye. The authors found that neither distraction nor the lack of visual awareness of the adapters could suppress the spiral MAE, though the effect was reduced in strength. However, their most relevant finding was that no spiral MAE was generated when attention was diverted from the unseen spiral adapters.

These different results may reflect the involvement of different visual mechanisms underlying CFS and crowding. In this context, Faivre et al. [59] compared the processing of facial expressions rendered invisible using crowding and CFS. The authors found that pictures of happy faces suppressed from awareness by crowding were processed sufficiently to bias subsequent preference judgments. The same stimuli suppressed with CFS did not bias the preference judgments, although they were processed such as to elicit perceptual priming. The lack of preference bias is in line with theories of binocular rivalry that states that drastic suppression at early stages of visual processing (e.g., subcortical structures and striate cortex) takes place, thus interfering with processing at higher visual areas [108, 109]. On the other hand, stimuli in crowded conditions biased subsequent preference judgments, suggesting that high-level processing is still preserved despite the lack of perceptual awareness.

There is indeed neuroimaging evidence that crowding occurs at different levels of visual analysis [49, 57, 58, 61]. Bi et al. [58], for example, investigated the effect of crowding on orientation-selective adaptation in human early visual areas and found that when attention was controlled orientation-selective fMRI adaptation in the striate cortex was not affected by crowding. However, the authors found that crowding weakened the orientation adaptation effect in V2 and V3, suggesting that crowding for orientation occurs beyond V1. On the other hand, Chen et al. [110] using event related potentials and fMRI found that the magnitude of crowding was strongly related with an early suppressive cortical interaction in V1, not present in higher cortical areas. Moreover, the authors found that spatial attention played an important role in generating such cortical suppression. These results provide evidence for an attention-based suppression at the level of V1 that contributes to crowding at early stage of visual analysis.

The interaction between awareness and attention found by Kaunitz et al. [33] may depend on the technique they used to suppress the awareness of the adapter. Indeed, it is likely that CFS operates at a low level of visual analysis and it might be more susceptible to attentional modulation. Indeed there is neuroimaging evidence that attentional load strongly modulates activity evoked in V1 by stimuli, whose awareness is suppressed with the CFS paradigm [111]. Thus, within this framework, the dissociation we found between awareness and attention may depend on the fact that in our case crowding occurs at a high level of motion processing, where attention does not modulate motion adaptation [75–77].

## CONCLUSIONS

For the first time we investigated the interplay between crowding and attention at a high level of motion processing. The results showed that conventional and phantom MAE are preserved in crowding, though slightly reduced in strength. When matching the strength of the conventional and the phantom MAE we did not find evidence for an effect of distraction. This result is in agreement with those reported in a series of studies by Morgan [75–77], who found that distracted attention does not impair the strength of the MAE. Moreover, our findings suggest that crowding at a high level of motion processing is not modulated by attention.

Further psychophysical and neuroimaging experiments will be necessary to better assess the role of attention at high levels of motion processing and the interplay between attention and awareness along the motion processing hierarchy. There are indeed plenty of examples in literature that show how attention can clearly bias the percept of directionally ambiguous, moving stimuli (e.g., attention-based motion [64, 65, 112, 113] or induce the MAE [114]). The present study contributes to the existent literature by illustrating how crowding affects both conventional and phantom MAE. Attention, on the other hand, seems to play only a minor role in the build-up of the motion aftereffect, as its effects on the conventional and phantom MAE were minor or non-existent.
